# A comprehensive review of the recent advances on predicting drug-target affinity based on deep learning

**DOI:** 10.3389/fphar.2024.1375522

**Published:** 2024-04-02

**Authors:** Xin Zeng, Shu-Juan Li, Shuang-Qing Lv, Meng-Liang Wen, Yi Li

**Affiliations:** ^1^ College of Mathematics and Computer Science, Dali University, Dali, China; ^2^ Yunnan Institute of Endemic Diseases Control and Prevention, Dali, China; ^3^ Institute of Surveying and Information Engineering West Yunnan University of Applied Science, Dali, China; ^4^ State Key Laboratory for Conservation and Utilization of Bio-Resources in Yunnan, Yunnan University, Kunming, China

**Keywords:** deep learning, drug-target affinity, dataset, representation, methods

## Abstract

Accurate calculation of drug-target affinity (DTA) is crucial for various applications in the pharmaceutical industry, including drug screening, design, and repurposing. However, traditional machine learning methods for calculating DTA often lack accuracy, posing a significant challenge in accurately predicting DTA. Fortunately, deep learning has emerged as a promising approach in computational biology, leading to the development of various deep learning-based methods for DTA prediction. To support researchers in developing novel and highly precision methods, we have provided a comprehensive review of recent advances in predicting DTA using deep learning. We firstly conducted a statistical analysis of commonly used public datasets, providing essential information and introducing the used fields of these datasets. We further explored the common representations of sequences and structures of drugs and targets. These analyses served as the foundation for constructing DTA prediction methods based on deep learning. Next, we focused on explaining how deep learning models, such as Convolutional Neural Networks (CNNs), Recurrent Neural Networks (RNNs), Transformer, and Graph Neural Networks (GNNs), were effectively employed in specific DTA prediction methods. We highlighted the unique advantages and applications of these models in the context of DTA prediction. Finally, we conducted a performance analysis of multiple state-of-the-art methods for predicting DTA based on deep learning. The comprehensive review aimed to help researchers understand the shortcomings and advantages of existing methods, and further develop high-precision DTA prediction tool to promote the development of drug discovery.

## 1 Introduction

Drug-target affinity (DTA) is a critical metric and the core of drug discovery. While the wet experiments have been used to calculate DTA with high accuracy, the time-consuming and laborious nature of these experiments can no longer meet the demands of modern drug screening, especially with the massive drug-target pairs. Fortunately, the emergence of computational methods for predicting DTA has accelerated the drug screening process, helping to shorten the drug development cycle and reduce the costs (Kairys et al., 2019; Abbasi et al., 2021; Xu et al., 2021; Zhang et al., 2023a).

At present, while there are non-machine learning methods available for computing DTA, such as FEP (Free-Energy Perturbation) (Jorgensen and Thomas, 2008) and MM/GBSA (or MM/PBSA) (Çınaroğlu and Timuçin, 2020), which can effectively estimate the binding free energy or affinity of drug-target, these methods not only demand a significant amount of computing resources, but also exhibit slow processing speeds when dealing with a large number of drug-target pairs. In contrast, data-driven machine learning methods offer fast processing speeds and high computational accuracy. The computational methods based on machine learning for predicting DTA can be classified into two categories: traditional machine learning methods and deep learning methods. Traditional machine learning methods employ linear regression, random forest regression, nearest neighbor regression, and support vector machine regression (Ballester and Mitchell, 2010; Li et al., 2015; Shar et al., 2016) to predict DTA. Although these methods perform well performance, they cannot automatically extract high-level hidden features from drugs and targets. With the emergence of deep learning models, DTA prediction methods based on deep learning (Öztürk et al., 2018; Wang et al., 2021a; Rube et al., 2022) can automatically extract high-level hidden features from the sequences and structures of drugs and targets, resulting in the improved performance compared to traditional machine learning methods. Except for a few methods that utilize deep learning to extract high-level features from target-target and drug-drug interaction networks (Dehghan et al., 2023; Rafiei et al., 2023), based on the different combinations of multiple modal features of drugs and targets, such as fingerprints, SMILES, two-dimensional molecular topology graphs, three-dimensional spatial structures, physicochemical properties, sequences, and contact maps, deep learning-based DTA prediction methods can be broadly divided into three categories: sequence-based, hybrid-based, and structure-based methods.

Sequence-based methods (Öztürk et al., 2018; Karimi et al., 2019; Wang et al., 2021a; Li et al., 2022b; Ghimire et al., 2022; Zhao et al., 2022; Gim et al., 2023; Jin et al., 2023; Kalemati et al., 2023; Ru et al., 2023; Zhou et al., 2024) aim to extract implicit sequence features from drug SMILES (Simplified Molecular Input Line Entry System) (Weininger, 1988) and target sequences using deep learning models. These methods leverage various sequence deep learning models such as Convolutional Neural Networks (CNNs) (LeCun et al., 2015), Recurrent Neural Networks (RNNs) (Zaremba et al., 2015), and Transformers (Vaswani et al., 2017). In the current sequence-based methods, 1D-CNN, RNNs, and Transformers are commonly used to extract high-level sequence features. On the other hand, 2D-CNN is employed to extract sequence features from a two-dimensional matrix composed of drugs or targets. For instance, DeepDTA (Öztürk et al., 2018) utilized a CNN module with three consecutive 1D convolutional layers to extract sequence features from drug SMILES and target sequences, respectively. SimCNN-DTA (Shim et al., 2021), on the other hand, employed 2D-CNN to predict DTA by utilizing the outer product between column vectors of two similar matrices representing drugs and targets. While CNNs effectively capture the local features from drug SMILES and target sequences, they may overlook long-range dependencies between atoms or amino acids. To address this issue, RNNs with memory functions can be utilized to extract long-range dependent features, as demonstrated in DeepAffinity (Karimi et al., 2019) and DeepCDA (Abbasi et al., 2020). However, CNNs and RNNs may not focus on the key features influencing drug-target interaction or provide interpretability for the model’s effectiveness. Some attention mechanisms (Vaswani et al., 2017) are employed to capture the key features (Zeng et al., 2021; Chen et al., 2022; Ghimire et al., 2022; Monteiro et al., 2022; Zhang et al., 2022; Zhao et al., 2022). For example, AttentionDTA (Zhao et al., 2022) utilized attention mechanisms to focus on subsequences within drug SMILES and target sequences that played a crucial role in affinity prediction. MRBDTA (Zhang et al., 2022) incorporated multi-head attention mechanisms, effectively capturing drug-target interaction sites and providing interpretational analysis for its effectiveness. CAPLS (Jin et al., 2023) employed the cross-attention mechanism to capture the mutual effect of protein-binding pocket and ligand. MT-DTA (Zhu et al., 2023b) built a variational autoencoders system with a cascade structure of attention model and CNNs to extract the implied high-level interactive features from target sequences and drug SMILES. Sequence-based methods have the advantage of easily obtaining the target sequences and drug SMILES data. These methods excel in processing sequence data swiftly, without demanding substantial computing resources, and exhibit a fine performance in predicting DTA based on the extracted high-level sequence features. Nevertheless, these methods overlook additional multimodal information related to targets and drugs, like topology graphs and 3D structures. It is important to note that structural information harbors crucial features that significantly influence DTA prediction. Disregarding this essential structural data may limit the accuracy, depth, and interpretability of understanding in predicting DTA. However, utilizing the structures of targets to enhance DTA faced limitations in the early states, as only a small portion of target sequences had known structures. Consequently, the exploration of hybrid-based methods emerged by incorporating the structural features of drugs into sequence-based approaches.

Hybrid-based methods (Karimi et al., 2021; Wang et al., 2021b; Zhang et al., 2021; Cheng et al., 2022; Li et al., 2022a; Lin et al., 2022a; Tian et al., 2022; Yang et al., 2022; Jiang et al., 2023; Pan et al., 2023; Wang et al., 2023a; Wang and Li, 2023; Xia et al., 2023; Yang et al., 2023; Zeng et al., 2023; Zhang et al., 2023b; Zhang et al., 2023a; Zhu et al., 2023a; Zhu et al., 2023c; Nguyen et al., 2022.) leverage deep learning models to extract sequence features from drug SMILES and target sequences, as well as the structural features from two-dimensional molecular topology graphs and three-dimensional structures of drug small molecules. These methods focus on integrating the structural features of drugs into sequence-based approaches. For the structures of drugs, tools like RDKit (Landrum, 2013) are commonly used to convert drug SMILES into the molecular graphs. GNN models (Xu et al., 2019) are employed for capturing the structural features of drugs. For instance, GraphDTA (Nguyen et al., 2021) utilized GCN and CNN to extract the structural features from drug molecular graphs and sequence features from target sequences, respectively. These extracted features were then combined as inputs and passed through fully connected layers to predict DTA. SAG-DTA (Zhang et al., 2021) incorporated a GCN with multiple self-attention graph pooling layers to extract the hidden features from drug molecular graphs. CNN was directly applied to the target sequences for learning high-level features. TDGraphDTA (Zhu et al., 2023c) introduced the transformer and diffusion to predict drug-target interactions using multi-scale information interaction and graph optimization. Hybrid-based methods combine the structural features of drugs with sequence-based approaches, enriching the features of drugs. Typically, GNN are employed to extract the drug structural features from molecular graphs converted from drug SMILES. These molecular graphs are relatively small and have minimal impact on the computational speed of the model. However, three-dimensional structural features of drugs are underutilized in hybrid-based methods. Furthermore, these methods completely overlook the structural features of target and make it difficult to provide explanatory analysis for the effectiveness of the model, leaving ample opportunity for performance enhancement. However, with the advent of AlphaFold (Jumper et al., 2021) and ColabFold (Kim et al., 2023), two target structural prediction tools, obtaining target structures has become less challenging. Consequently, there is a growing interest in methods that utilize the structures of drugs and targets for predicting DTA. Structure-based methods are gaining increased attention from researchers in this context.

Structure-based methods (Gomes et al., 2017; Stepniewska-Dziubinska et al., 2018; Zhang et al., 2019; Jiang et al., 2020; Seo et al., 2021; Shen et al., 2021; Lin et al., 2022b; Ma et al., 2022; Lu et al., 2023; Wu et al., 2024) employ deep learning models like GNN and 3D Convolutional Neural Network (3D-CNN) to extract implicit structural features from the molecular graphs of drugs and targets or the 3D structures of drug-target complexes. Using GNNs (Li et al., 2021; Yuan et al., 2021; Chu et al., 2022; Jiang et al., 2022; Liao et al., 2022; Pandey et al., 2022; Bi et al., 2023; Wang et al., 2023b; Zhang et al., 2023d; Zhang et al., 2023c; Ma et al., 2023; Mekni et al., 2023; Tsui et al., 2023; Tian et al., 2024), the molecular graphs of drugs and targets are fed into GNN to obtain the structural features. For example, PSG-BAR (Pandey et al., 2022) served as an example where a contact map was generated based on the 3D structure of target. Target graph was then constructed using the contact map, and the structural features were extracted using RGAT. For drug, the graph was generated based on its structural file, and RGAT was also employed to extract high-level features. AttentionMGT-DTA (Wu et al., 2024) represented drugs and targets by a molecular graph and binding pocket graph, respectively. Graph transformer module was utilized to extract the structural features of drugs and binding pockets. WGNN-DTA (Jiang et al., 2022) constructed protein and molecular graphs through sequence and SMILES that can effectively reflect their structures. Weighted graph neural networks were used to extract the structural features of molecules and proteins for predicting DTA. On the other hand, 3D-CNN-based methods (Zheng et al., 2019; Kwon et al., 2020; Liu et al., 2021; Wang et al., 2022) directly take the 3D structure of drug-target complex as input and use the extracted spatial features of complex as input for the FC network to predict DTA. For example, AK-Score (Kwon et al., 2020) employed the ensemble of multiple independently trained networks that consisted of multiple channels of 3D-CNN layers to predict the binding affinity of a complex. Sfcnn (Wang et al., 2022) converted drug-target complex into 3D grids for CNN training to extract the structural features. Structure-based methods offer effective utilization of the structural features of drugs and targets, yielding impressive performance. They are especially valuable for providing explanatory analyses that shed light on the model’s effectiveness, thereby facilitating research into DTA prediction methods and promoting wider application of these models. However, it is important to acknowledge some limitations. One such limitation is the reliance on tools like AlphaFold to obtain the target structures. While AlphaFold has shown higher accuracy in predicting the structures of monomeric proteins, its performance in predicting the structures of other proteins still requires optimization. Additionally, structure-based methods extract structural features from protein structure graphs, which can be computationally demanding and result in slower processing speeds.

In this review, we aimed to highlight the crucial significance of precise DTA prediction, followed by a comprehensive overview of the universal datasets and widely used representation methods for the sequences, structures, and complexes of drugs and targets. We then focused on the widespread application of popular deep learning techniques in DTA prediction. Our goal was to provide a comprehensive overview of datasets, representation, methods, and deep learning techniques for predicting DTA. By doing so, we intend to empower researchers to effectively utilize these resources in developing innovative DTA prediction methods, thereby providing essential support for drug discovery, design, and repurposing endeavors. The main contributions of this review can be summarized as follows:

(1) A comprehensive statistical analysis has been carried out on datasets, representations, model architectures, and performance evaluation of state-of-the-art methods based on deep learning for predicting DTA.
(2) Elaboration on the extraction process of crucial implicit features from diverse modalities of drugs and targets using cutting-edge deep learning technologies like CNN, RNN, GNN, and Transformer.
(3) An in-depth analysis of the strengths and limitations of advanced deep learning methods for predicting DTA is conducted from three perspectives: sequence, hybrid, and structure. This analysis serves as a foundation for researchers to develop novel and more accurate tools for DTA prediction.

## 2 Dataset

A high-quality dataset of drug-target binding affinity serves as the fundamental basis for the development of computational methods that leverage deep learning for predicting DTA. Currently, the most widely used datasets for DTA prediction include PDBbind (multiple versions) (Wang et al., 2005), Davis (Davis et al., 2011), KIBA (Tang et al., 2014), BindingDB (Liu et al., 2007), and Metz (Metz et al., 2011). To supplement these universal affinity datasets, UniRef (Suzek et al., 2015), UniProt (The et al., 2021), Protein Data Bank (PDB) (Berman, 2000), STITCH (Kuhn et al., 2007), and ZINC (Irwin and Shoichet, 2006) can provide additional sequences and structures for drugs and targets that may be missing.

### 2.1 Statistical analysis of commonly used datasets for DTA prediction

**FIGURE 1:**
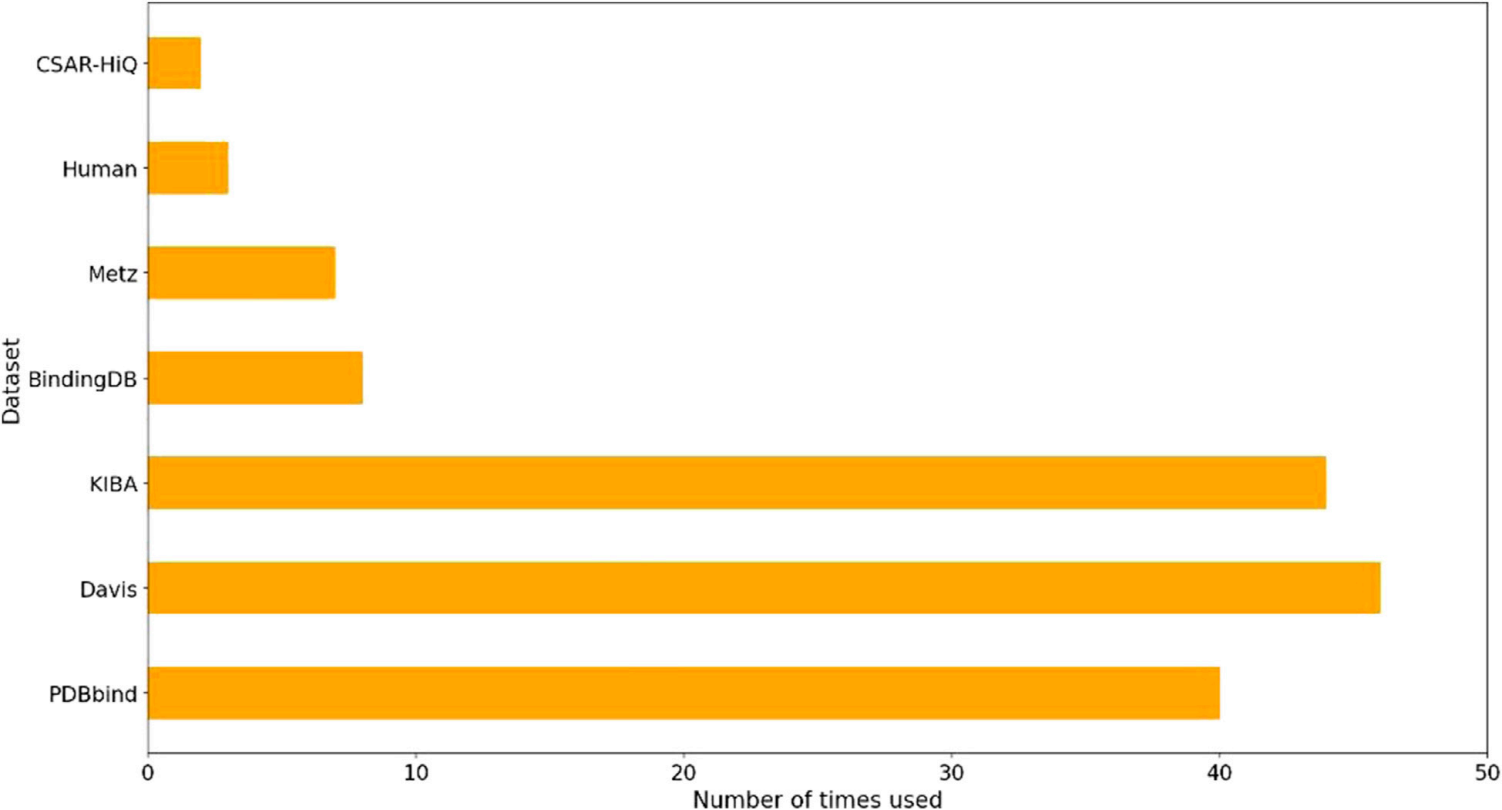
Statistics on the usage of the datasets for predicting DTA based on deep learning.

We performed a comprehensive statistical analysis on the datasets utilized in nearly 80 references on drug-target affinity cited in this review to assess their usage. The results of our analysis, as depicted in Figure 1, revealed that PDBbind, Davis, KIBA, BindingDB, and Metz were the five most frequently employed datasets. Among these, PDBbind and BindingDB were primarily utilized for deep learning methods based on hybrid or structure. These two datasets offer comprehensive sequence and structural data for drugs and targets. On the other hand, Davis, KIBA, and Metz were predominantly employed for sequence-based deep learning methods, although some hybrid or structure-based deep learning methods also utilized them. It is worth mentioning that the structures of targets in Davis, KIBA, and Metz were sourced from the PDB database.

### 2.2 Introduction to commonly used datasets for DTA prediction

In this section, we provide a detailed introduction to the five most frequently used datasets: PDBbind, Davis, KIBA, BindingDB, and Metz. In addition, we will also introduce the ToxCast dataset (Feng et al., 2019), which is commonly used in multi-task prediction methods.

#### 2.2.1 PDBbind

PDBbind dataset comprises four commonly used versions: 2013, 2016, 2018, and 2020. Each version of the dataset consists of two distinct parts: the general set and the refined set (Table 1). To illustrate, let’s consider the PDBbind dataset (version 2016), which includes a total of 13,283 samples: 9,226 samples for the general set and 4,057 samples for the refined set. The refined set is obtained based on the quality protocols, including measured resolution and experimental precision. This process ensures the exclusion of ligands, ternary complexes, or steric hindrance complexes with resolutions above 2.5 Å, R factors exceeding 0.25, instances of covalent bonding, and complexes lacking reported binding affinities in terms of Kd (dissociation constant) or Ki (inhibitor constant), or falling outside the necessary range (Kd < 1 pM). Each sample represents a drug-target pair labeled with affinity value known as the dissociation constant (Kd). Notably, each sample provides drug SMILES and target sequence, as well as the 3D structure of target and pocket information related to drug-target binding. Thanks to the extensive sequence and structural information available for drugs, targets, and pockets in PDBbind dataset, it has become widely recognized as a universal dataset for predicting DTA in sequence, hybrid, and structure-based deep learning methods. Furthermore, it is worth mentioning that the CASF series datasets used to test the performance of models in certain studies (Stepniewska-Dziubinska et al., 2018; Wang et al., 2022), such as CASF-2013 (Li et al., 2014), CASF-2016 (Su et al., 2019), are the core sets derived from the corresponding refined sets of PDBbind datasets.

**TABLE 1:** Statistic of commonly used PDBbind dataset with different versions.

Version	Total number of samples	General set	Refined set
2013	11,184	8,225	2,959
2016	13,283	9,226	4,057
2018	16,126	11,663	4,463
2020	19,443	14,127	5,316

#### 2.2.2 Davis and KIBA

Davis dataset (Table 2) comprises 68 compounds and 442 proteins, generating a total of 30,056 compound-protein affinity samples, each labeled with the dissociation constant (Kd). It is worth noting that all drug-target pairs that can not be experimentally measured for bioactivity are assigned a bioactivity value of 10 μM (corresponding to a p*K*_d_ of 5) in Davis dataset. But the number of data points within this range is very large. Consequently, some methods have chosen to remove the data points with a bioactivity value of 10 μM from the Davis dataset, thereby creating what is known as the Filtered Davis dataset (Rifaioglu et al., 2021). KIBA dataset (Table 2) includes 246,088 interaction pairs of samples derived from 467 proteins and 52,498 compounds. Notably, KIBA contains three types of labels: inhibition concentration 50 (IC50), dissociation constant (Kd), and inhibition constant (Ki). Due to their focus on only providing the sequences of drugs and targets, Davis and KIBA are predominantly utilized in sequence-based deep learning methods. Nonetheless, a few hybrid or structure-based approaches have also been successfully employed using these datasets. It is important to note that while Davis and KIBA do not include the 3D structures of targets and drugs, they can be accessed by downloading them from the PDB and ZINC databases, respectively.

**TABLE 2:** Detailed information on datasets Davis, Filtered Davis, and KIBA.

Dataset	Compounds	Proteins	Total number of samples
Davis	68	442	30,056
Filtered Davis	68	379	9,125
KIBA	52,498	467	246,088

#### 2.2.3 BindingDB

BindingDB dataset is primarily composed of drug-target pair samples sourced from some scientific literatures, encompassing four different types of affinity labels: IC50, Kd, Ki, and EC50 (median effect concentration). Table 3 displays the number of drugs, targets, and drug-target interaction pairs in each label category. Notably, BindingDB provides drug SMILES and target sequences, while their structures can be obtained from PDB and ZINC databases, respectively. This comprehensive information enables BindingDB to be widely utilized in sequence, hybrid, and structure-based methods, typically to evaluate the generalization performance of DTA prediction methods.

**TABLE 3:** Details of BindingDB dataset.

Dataset	Label	Drugs	Targets	Total number of samples
BindingDB	IC50	265,627	2,793	376,751
	Kd	5,895	812	12,589
	Ki	93,437	1,619	144,525
	EC50	31,970	513	37,896

#### 2.2.4 Metz

Metz dataset comprises 1,423 drugs and 170 targets, resulting in a total of 35,259 drug-target pairs. Each pair is labeled with an affinity value represented by Ki (in the form of p*K*_i_ value). Furthermore, the relationship between drugs and targets can be accessed from the STITCH database, which consolidates diverse chemical and protein networks.

#### 2.2.5 ToxCast

Toxcast is a toxicology research dataset derived from high-throughput *in vitro* screening of chemicals, primarily measuring AC50, which represents the concentration at half of the maximum activity. This dataset has a large scale, covering different types of proteins, and contains qualitative results from more than 600 experiments involving over 8,000 compounds. With around 530,000 observations of drug-target pairs and over 600 labels, it is well-suited for multi-task prediction. Its subsets are frequently utilized for case studies or generalization performance testing of DTA methods.

### 2.3 Introduction to supplementary used databases for DTA prediction

#### 2.3.1 Uniprot

Uniprot database (The et al., 2021) is a sequence database designed specifically for proteins that contains approximately 220 million protein sequences and related annotation information on the biological functions of proteins. It has the ability to add new protein entries, as well as supplement and update publicly available annotation information, and is widely regarded as the protein database with the most extensive collection and comprehensive annotation information.

#### 2.3.2 PDB

PDB database (Berman, 2000) is the premier collection of 3D structures for biological macromolecules, such as proteins, nucleic acids, etc., which contains the 3D structures of all resolved proteins. In addition to annotating the 3D structural information of proteins, PDB also provides various file types for downloading and visualizing the 3D structures of proteins.

#### 2.3.3 STITCH

STITCH database (Kuhn et al., 2007) is a valuable resource that includes information on interactions between 43,000 compounds and 9,643,763 proteins from 2,031 species. It shares protein interaction data with the STRING database (https://cn.string-db.org/), making it an important database for studying compound sequences. Each interaction in STITCH database is assigned a score value, which represents the affinity or probability of the interaction between a compound and a protein. STITCH also provides information on compounds that are similar to the drug of target, along with their similarity scores.

#### 2.3.4 ZINC

ZINC (Irwin and Shoichet, 2006) is a free commercial database used for virtual screening of compounds, which provides access to 3D structures of over 230 million molecules. It offers multiple docking program interfaces, user-defined molecular operations, and web-based database search and browsing capabilities.

## 3 Representation

### 3.1 Sequence representation

Drug SMILES and target sequences are composed of different characters. Therefore, they are commonly encoded using one-hot encoding or label encoding in sequence and hybrid-based methods. Their sequence features are extracted using CNN, RNN, or Transformer. In structure-based methods, the extracted features from sequences are utilized as node features in the graphs of drugs and targets. In addition, traditional sequence features such as molecular fingerprint, position-specific score matrix (PSSM) (Altschul, 1997), and Hidden Markov Matrix (HMM) (Remmert et al., 2012) are also widely employed in DTA prediction.

### 3.2 Structure representation

For drug, the structure representation often involves graph. One common type of the drug graph is based on the drug SMILES, which can be converted using RDKit tool. Another type of the drug graph is based on the 3D structural file, where atoms serve as vertices and bonds act as edges. Node features in the drug graph can be derived from the physical-chemical properties of atoms or extracted from drug SMILES using deep learning techniques.

For target, the secondary structural information can be obtained directly from the relevant file of target and is widely employed in traditional machine learning and deep learning methods. The tertiary structural graph of target can be roughly categorized into two types: contact map and spatial topology graph. Contact map is created based on the sequence or tertiary structure of target, generating a map of interaction between amino acids. Structural features of target can be extracted directly from the contact map using CNN models. Alternatively, the contact map can be converted into a target graph, allowing the use of GNNs to extract structural features. Spatial topology graph of target is constructed based on the 3D structural file. Nodes in the graph represent amino acids, typically carbon α atoms, and edges are formed based on distance thresholds, such as Euclidean distance between carbon α atoms.

### 3.3 Interaction network graph representation of drug-target complex

In DTA prediction, the interaction between a drug and its target is often represented as a graph. This involves extracting interaction features using GNN. The construction of the interaction network graph is based on the 3D structure of drug-target complex. To create the graph, the atoms of drug and the carbon atom of amino acid in target (typically the carbon α atom) are selected as the vertices of graph. The Euclidean distance between each atom and the carbon atom is then calculated. If the distance is less than or equal to a specified threshold (usually set to 8 Å or 10 Å), an edge is created to connect the corresponding atom to the amino acid. Any atoms and amino acids that did not participate in the construction of the interaction graph are excluded. This process results in an interaction network graph that represents the drug-target complex, which can be used for analysis and prediction of DTA.

### 3.4 3D structural spatial grid representation of drug-target complex

While the interaction network graph of drug-target complex can provide valuable information about the structural features, some atoms and amino acids are ignored. As a result, deep learning methods that utilize a complete 3D structural spatial grid representation of drug-target complex are widely used. The 3D structural spatial grid representation of the complex is composed of the spatial coordinates of all atoms, and 3D-CNN is used to extract the spatial structural features from the complex’s 3D structure.

## 4 Drug-target affinity prediction methods based on deep learning

Currently, computational methods for predicting DTA using deep learning can be broadly categorized into three groups based on the progression from sequence to structure: sequence-based, hybrid-based, and structure-based methods. In the following chapters, we will provide a comprehensive overview of the feature extraction process for each category.

### 4.1 Sequence-based deep learning methods

**FIGURE 2:**
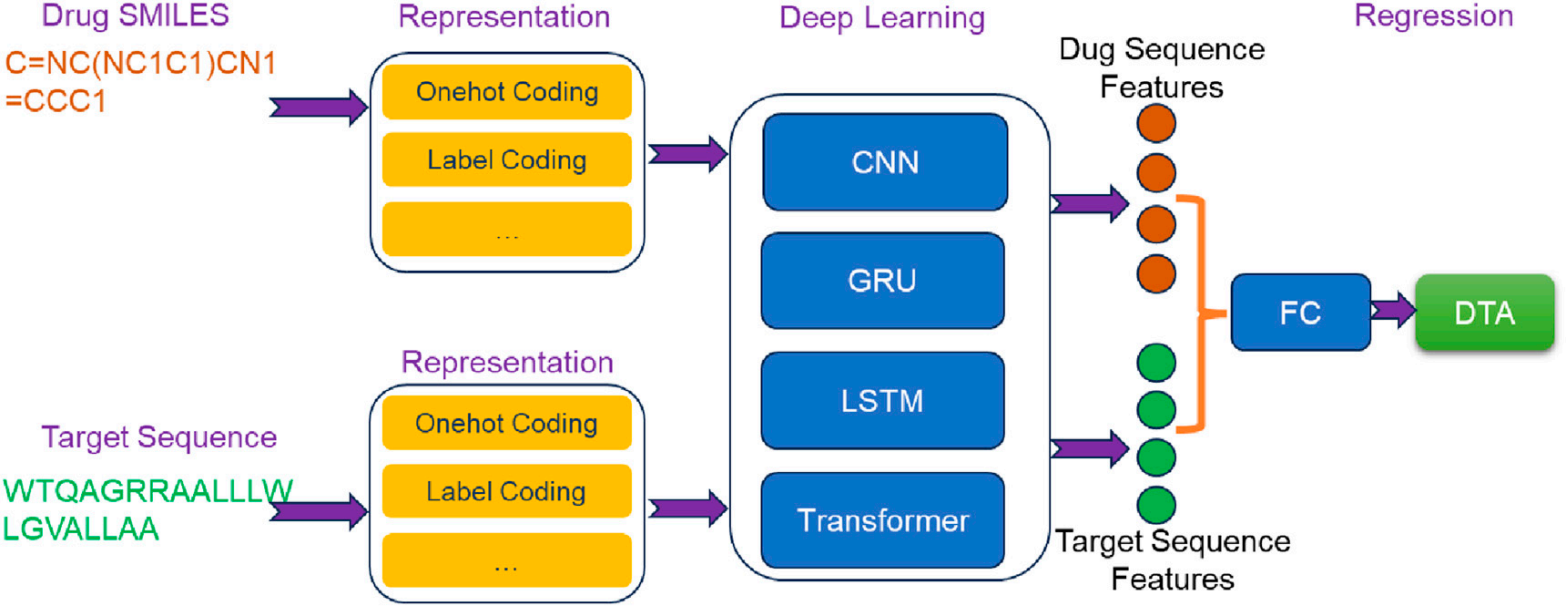
The overview architecture of sequence-based deep learning methods for predicting DTA.

Sequence-based deep learning methods (Figure 2) utilize drug SMILES and target sequences as input. These methods employ various deep learning techniques, including CNN, RNN, Transformer, and attention mechanisms, to extract essential features from the input sequences. In the following sections, we will provide an overview of some classic sequence-based methods.

#### 4.1.1 DeepDTA

In DeepDTA (Öztürk et al., 2018), drug SMILES and target sequences were encoded as label encodings and used as inputs. The sequence feature extraction was conducted by two independent CNN blocks, each comprising three 1D convolutional layers. Drug SMILES and target sequences, were separately processed through the embedded layers and passed into their respective CNN blocks. This allowed for the extraction of high-level sequence features from drugs and targets. Subsequently, the extracted sequence features were concatenated and fed into a three-layer FC network to predict DTA. DeepDTA not only showed superior performance compared to traditional machine learning methods, but also enabled automatic extraction of sequence features and end-to-end DTA prediction. This contributed to the transition from traditional machine learning methods to deep learning methods in the field of DTA prediction.

#### 4.1.2 DeepCDA

In the architecture of DeepCDA (Abbasi et al., 2020), drug SMILES and target sequences were used as inputs. Initially, both drug SMILES and target sequences underwent encoding via coding layers. The encoded representations were then separately fed into identical feature extraction networks. Each feature extraction network consisted of two components: a CNN block and an LSTM block. CNN block comprised three convolutional layers, responsible for extracting short-distance features from the sequences. These short-distance features were subsequently inputted into a multi-layer LSTM block to capture long-distance dependent features. By combining CNN and LSTM, DeepCDA effectively considered local and long-range dependent features of the sequence. To further extract crucial information influencing drug-target interaction, a bidirectional attention mechanism was employed to fuse the extracted sequence features. This fusion process enabled comprehensive feature mining that accounts for the interaction between drugs and targets. Finally, the fused features were fed into a FC layer to predict DTA.

#### 4.1.3 AttentionDTA

AttentionDTA (Zhao et al., 2022) took drug SMILES and target sequences as input, which were encoded using label encoding. A character embedding layer was inserted between the label encoding layer and the feature extraction block to convert drug SMILES and target sequences into embedding matrices. These matrices were then passed through a CNN block consisting of multiple 1D-CNN layers to extract implicit sequence features. To capture the non-covalent interactions between the atoms of drug and the amino acids of target, AttentionDTA incorporated a bilateral multi-head attention mechanism. This mechanism took the features extracted by the CNN block as input, allowing it to capture the interaction information that affected drug-target interaction. The resulting key interaction information was subsequently fed into a multi-layer perceptron (MLP) for DTA prediction.

### 4.2 Hybrid-based deep learning methods

**FIGURE 3:**
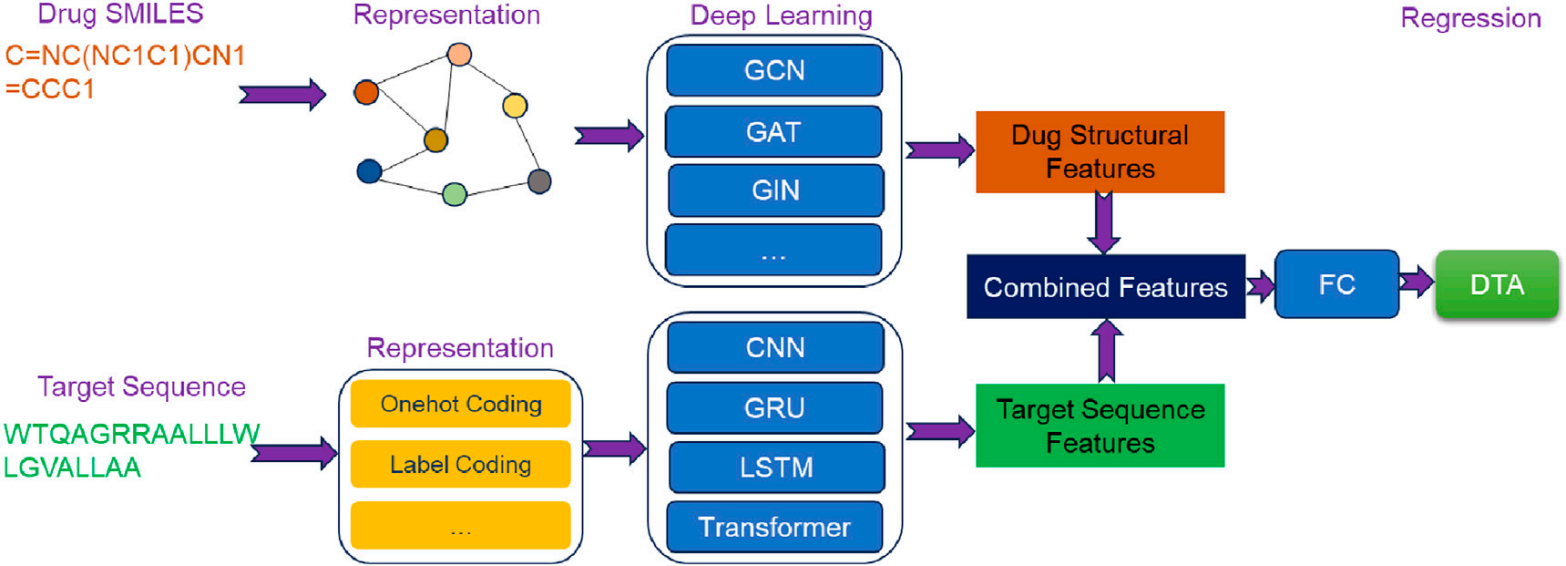
The overview architecture of hybrid-based deep learning methods for predicting DTA.

Hybrid-based deep learning methods (Figure 3) have been at the forefront of utilizing the structural features of drugs. The process begins by obtaining the graph representation of drug directly from its SMILES using RDKit tool. Subsequently, GNN is employed to learn implicit high-level structural features from the graph. Finally, these extracted structural features are combined with the sequence features of target to predict DTA. These methods effectively integrate the sequence and structural information to enhance the performance.

#### 4.2.1 GraphDTA

GraphDTA (Nguyen et al., 2021) was a representative hybrid-based deep learning method for predicting DTA. It leveraged the structural features of drugs and the sequence features of targets. Initially, drug SMILES was converted into a molecular graph using the RDKit tool. Subsequently, a three-layer GNN was employed to extract the structural features. As for target, the sequence underwent label encoding and embedding layers before being inputted into a convolutional block comprising three 1D-CNN layers to learn sequence features. Finally, the extracted structural features of drugs and the sequence features of targets were combined and fed into a FC network with multiple layers to estimate DTA.

#### 4.2.2 MGraphDTA

MGraphDTA (Yang et al., 2022) took a similar approach by leveraging the structures of drugs and target sequences. However, it enhanced the global structural features extraction by employing a deeper multi-scale GNN (MGNN). This allowed for a comprehensive understanding of the global relationships between atoms in drug and captured various features within the structure of drug. Simultaneously, multi-scale CNN (MCNN) was applied to extract multi-scale features from target sequences. Following this, the multi-scale features from the structures of drugs and target sequences were separately fused, and the resulting fused features were concatenated to form a combined representation of drug-target pair. Finally, the combined representation was fed into MLP to predict DTA.

#### 4.2.3 ColdDTA

Deep learning methods have exhibited promising performance on randomly split public datasets, but their performance tends to significantly decrease when applied to practical scenarios. To address this issue, ColdDTA (Fang et al., 2023) utilized the structural knowledge of drugs and target sequence information to enhance the model’s generalization performance by data augmentation and attention-based feature fusion techniques. The construction process of ColdDTA was as follows: firstly, a new drug-target pair was generated by removing a subgraph from the original graph of drug. Next, the structural features of drug and the sequence features of target were extracted using GNN and CNN, respectively. These extracted features were then fused via an attention-based fusion block to better capture the interaction mechanism between drug and target. Finally, the fused features were inputted into MLP to predict DTA.

### 4.3 Structure-based deep learning methods

**FIGURE 4:**
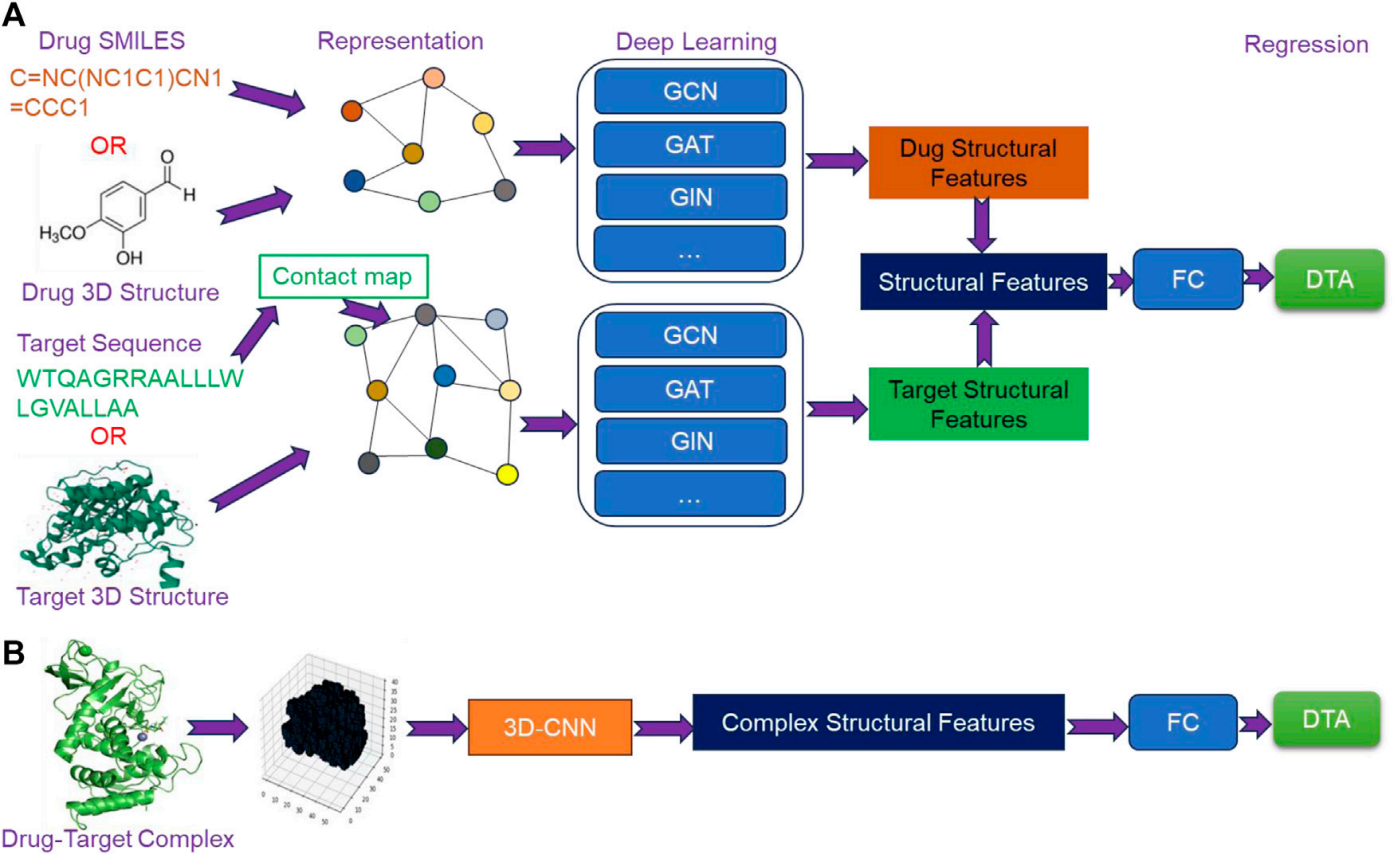
The overview architecture of structure-based deep learning methods. **(A)** The extraction of structural features from molecular graphs of drugs and targets using Graph Neural Networks (GNN), and **(B)** the extraction of structural features of drug-target complexes from their 3D structures using 3D Convolutional Neural Networks (3D-CNN).

Currently, structure-based deep learning methods for predicting DTA can be broadly categorized into two types. The first type involves extracting structural features from the molecular graphs of drugs and targets using GNN, followed by fusing the extracted features to predict DTA using a FC network (Figure 4A). The second type is based on 3D structures of drug-target complexes, where high-level structural features are extracted using 3D-CNN to predict DTA (Figure 4B). With the emergence of AlphaFold and ColabFold, obtaining the structures of targets has become more feasible. Furthermore, the rapid development of GNN and 3D-CNN has provided critical support for extracting structural features. As a result, structure-based methods have garnered increasing attention from researchers.

#### 4.3.1 GSAML-DTA

GSAML-DTA (Liao et al., 2022) employed a hybrid network model combining GNN and GAT to extract structural features from drugs and targets. The process began by converting drug SMILES and target sequences into drug molecular graphs and contact maps, respectively, using different tools. Subsequently, drug molecular graphs and contact maps were separately inputted into the hybrid network model of GNN-GAT with an attention mechanism to extract structural features of drugs and targets. Following this, the extracted features were concatenated and further optimized through an interactive information module. Finally, the optimized features were fed into a FC network to predict DTA. By leveraging the GNN-GAT hybrid network model with attention mechanism and the interactive module, GSAML-DTA aimed to enhance the accuracy of DTA prediction.

#### 4.3.2 HGRL-DTA

HGRL-DTA (Chu et al., 2022) utilized a hierarchical graph representation learning model for predicting DTA. This model established a hierarchical graph framework where the drug-target binding affinity data was represented as an affinity graph, with drugs and targets serving as vertices within the graph. Simultaneously, drugs and targets were represented as molecular graphs, respectively. To begin, GNN was employed to learn global-level affinity relationship within the affinity graph. Additionally, GNN was also used to separately capture the local chemical structural features of drugs and targets. Through a message propagation mechanism, the learned hierarchical graph information was integrated, and the structural features of drugs and targets were refined using GCN. Finally, these extracted structural features of drugs and targets were combined and inputted into a FC network to predict DTA. By leveraging the hierarchical graph setup, GNN-based representation learning, and message propagation mechanism, HGRL-DTA aimed to improve the accuracy of DTA prediction.

#### 4.3.3 MSGNN-DTA

MSGNN-DTA (Wang et al., 2023b) employed a multi-scale graph construction approach to capture the structural features of drugs and targets from multiple perspectives. For drugs, two types of graphs were constructed. Firstly, an atomic level graph was generated using RDKit tool based on drug SMILES. In this graph, atoms were represented as vertices, chemical bonds between atoms were represented as edges, and the topology was represented by a two-dimensional matrix. Secondly, a motif level graph was created by considering certain motifs (e.g., benzene rings) as vertices, with edges indicating the presence of chemical bond connections between motifs. Regarding target, target sequence was converted into a contact map using ESM-1b (Rives et al., 2021). Additionally, a weight map was constructed based on WGNN-DTA (Jiang et al., 2022). In weight map, residues served as vertices, interactions between residues served as edges, and weights of edges were probability values. To obtain multi-scale topological feature representations, GNN was utilized to extracted high-level structural features from the atomic level graphs and motif level graphs of drugs, as well as the weight graphs of targets. Subsequently, an attention mechanism was employed to fuse the multi-scale structural features and generate a join feature representation. The joint feature representation was then inputted into a multi-layer FC network for DTA prediction.

#### 4.3.4 Sfcnn

Aside from utilizing GNN to extract the structural features from molecular graphs of drugs and targets, there are some methods that use 3D-CNN to extract the structural features from drug-target complexes. One such method was Sfcnn, which employed 3D-CNN to generate a score function for DTA prediction. To begin, the drug-target complex was transformed into a 3D grid representation. This grid served as input to 3D-CNN, which learned high-level structural features. Finally, multiple density layers were applied to the extracted features for DTA prediction.

## 5 Performance analysis of multiple state-of-the-art methods based on deep learning

### 5.1 Common performance evaluation metrics

In this review, predicting drug-target affinity is a regression task, and commonly used performance evaluation metrics of the model include Mean Absolute Error (*MAE*), Mean Square Error (*MSE*), Root Mean Square Error (*RMSE*), Pearson Correlation Coefficient (*PCC*), Spearman ( ρ ), Concordance Index (*CI*), and *R* ^2^.

*MAE* Eq. 1 is used to measure the mean absolute error between prediction value and actual value. It reflects the size of actual prediction error.

$$MAE=\frac{1}{n}\sum_{i=1}^{n}\left|y_{i}-\hat{y_{i}}\right|,\in \left[0,+\right.\infty )$$ (1)

*MSE* Eq. 2 and *RMSE* Eq. 3 are often used to measure the deviation between prediction value and actual value. It is a measure of accuracy used to compare the prediction errors of different models for specific dataset and measure the error rate of the regression model. For *MAE*, *MSE*, and *RMSE*, the smaller their values are, the better effect of the model is.

$$MSE=\frac{1}{n}\sum_{i=1}^{n}{\left(y_{i}-\hat{y_{i}}\right)}^{2}$$ (2)

$$RMSE=\sqrt{\frac{1}{n}\sum_{i=1}^{n}{\left(y_{i}-\hat{y_{i}}\right)}^{2}},\in \left[0,+\right.\infty )$$ (3)

$R^{2}$ Eq. 4 is mainly used to measure how well the prediction value fits the actual value. When our model does not have any deficiencies, $R^{2}$ will get the maximum value of 1. If $R^{2}$ is 0, our model is equal to the baseline model. When $R^{2}$ is less than 0, it means that our model is not as good as the baseline model.

$$R^{2}=1-\frac{\sum_{i=1}^{n}{\left(y_{i}-\hat{y_{i}}\right)}^{2}}{\sum_{i=1}^{n}{\left(y_{i}-\bar{y}\right)}^{2}},\in \left[0,1\right]$$ (4)

In formula Eqs 1–4, n is the number of samples, $y_{i}$ is the vector of actual value, $\hat{y_{i}}$ is the precdition vector, and $\bar{y}$ is the average value of all actual values $y_{i}\;\left(1\leq i\leq n\right)$ .

*PCC* Eq. 5 is used to measure the mutual relationship (linear correlation) between two variables *X* and *Y*, and its range is [−1, 1]. *PCC* is widely used in academic research to measure the strength of the linear correlation between two variables. *Cov* (*X*, *Y*) represents the covariance of two variables *X* and *Y*. $\sigma_{X}$ is the standard deviation of *X*. If $\rho_{XY}>0$ , it means that *X* and *Y* are positively correlated; $\rho_{XY}<0$ , *X* and *Y* are negatively correlated; $\rho_{XY}=0$ , *X* and *Y* are not correlated.

$$\rho_{XY}=\frac{cov\left(X,Y\right)}{\sigma_{X}\sigma_{Y}},\in \left[-1,1\right]$$ (5)

Spearman Eq. 6 is a nonparametric measure of the dependence of two variables. n is the number of samples. The difference between prediction value and actual value of each group is $d_{i}\;\left(1\leq i\leq n\right)$ . The closer value of correlation coefficient ρ is to +1 or −1, the stronger correlation between two variables.

$$\rho =1-\frac{6\sum d_{i}^{2}}{n\left(n^{2}\right)}$$ (6)

*CI* Eq. 7 is used to evaluate the prediction accuracy of the model. Where $b_{i}$ is the prediction value for the larger affinity $\delta_{i}$ , $b_{j}$ is the prediction value for the smaller affinity $\delta_{j}$ , Z is a normalization constant. For function $\varphi \left(x\right)$ , it is 1 if the value of *x* is greater than 0, 0.5 if the value of *x* is equal to 0, and 0 if the value of *x* is less than 0.

$$CI=\frac{1}{Z}\sum_{\delta_{i}>\delta_{j}}\delta \left(b_{i}-b_{j}\right)\;\left(1\leq i,j\leq n\right)$$ (7)

### 5.2 Performance analysis of multiple state-of-the-art methods based on PDBbind, KIBA, and Davis datasets

**FIGURE 5:**
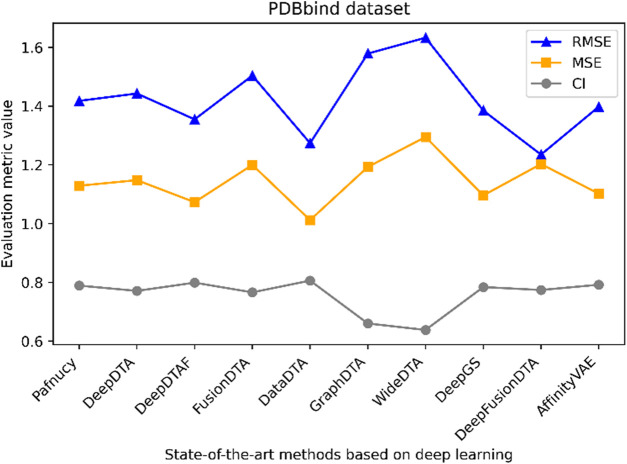
Performance analysis of multiple state-of-the-art methods based on PDBbind dataset. The general set and refined set are used as the training dataset, while the core set serves as the test dataset. The evaluation metric values of these methods in the figure are sourced from References (Wang et al., 2023a; Zhu et al., 2023a).

**FIGURE 6:**
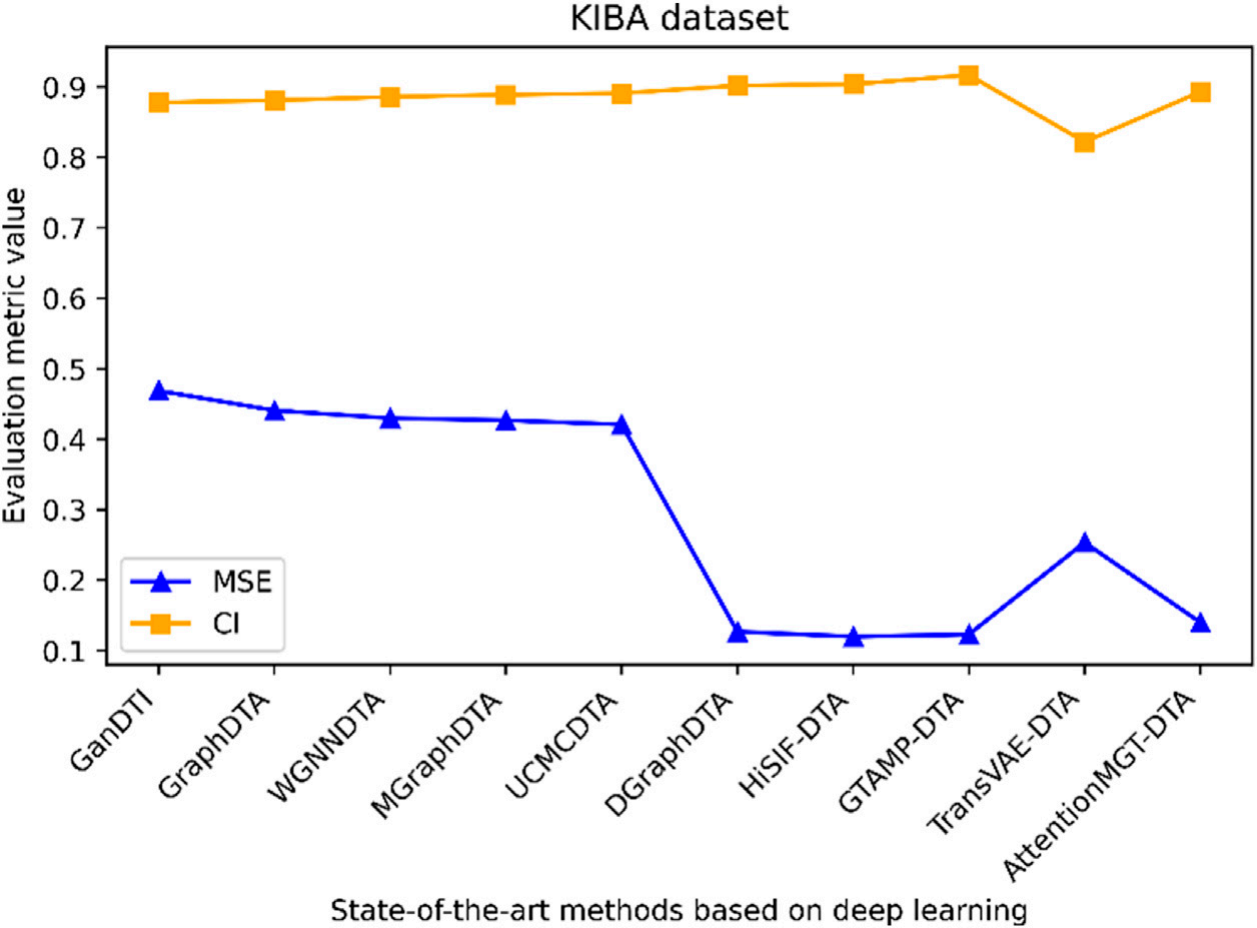
Performance analysis of multiple state-of-the-art methods based on KIBA dataset. The evaluation metric values of these methods in the figure are sourced from References (Bi et al., 2023; Xia et al., 2023; Tian et al., 2024; Wu et al., 2024; Zhou et al., 2024).

**FIGURE 7:**
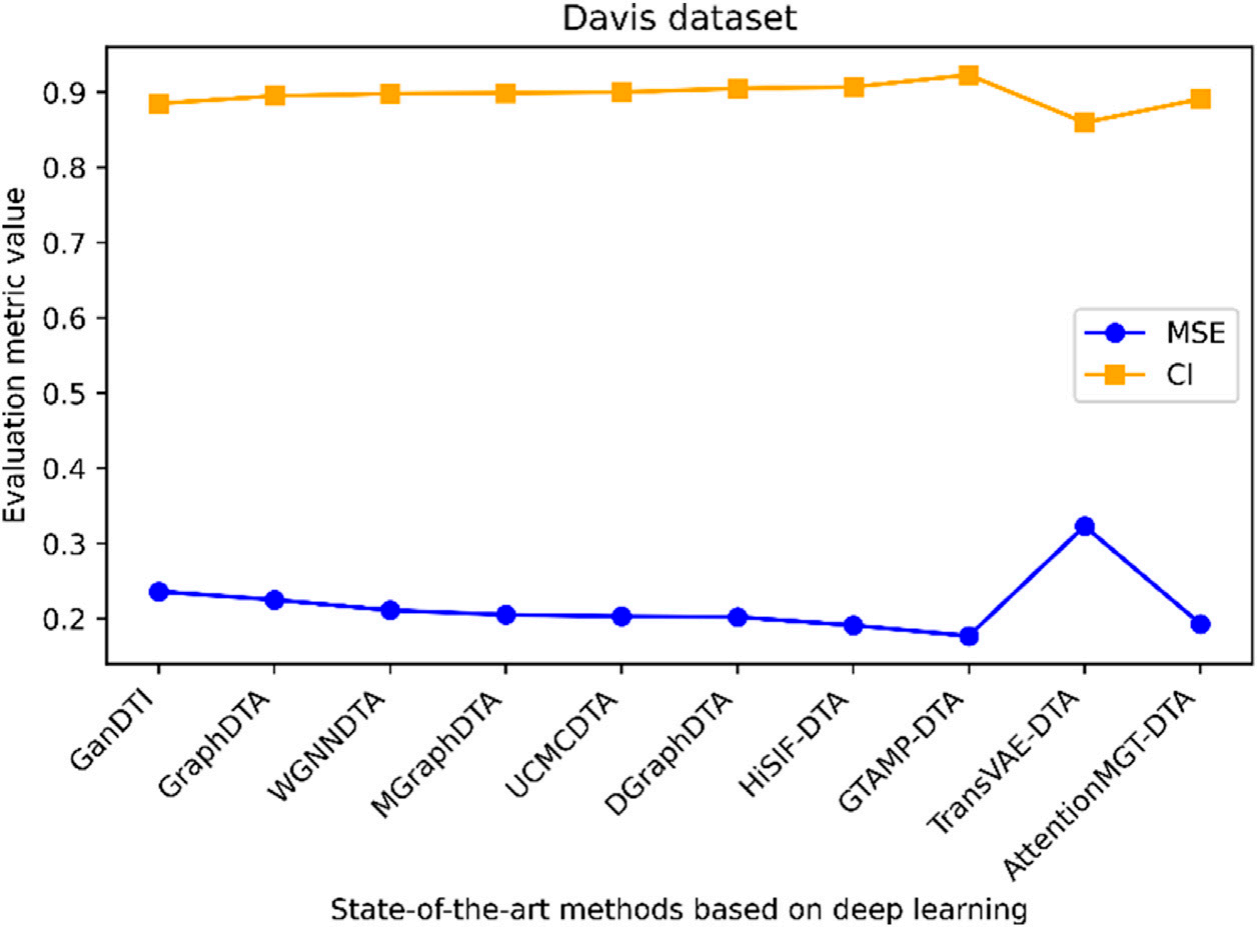
Performance analysis of multiple state-of-the-art methods based on Davis dataset. The evaluation metric values of these methods in the figure are sourced from References (Bi et al., 2023; Xia et al., 2023; Tian et al., 2024; Wu et al., 2024; Zhou et al., 2024).

Figure 1 highlights PDBbind, KIBA, and Davis datasets as commonly used datasets for predicting DTA using deep learning. We summarized the performance evaluation metrics values of several state-of-the-art methods on PDBbind, KIBA, and Davis datasets, as reported in recently published literatures (Wang et al., 2023a; Zhu et al., 2023a; Bi et al., 2023; Xia et al., 2023; Tian et al., 2024; Wu et al., 2024; Zhou et al., 2024), without considering the specific partitioning of the corresponding datasets by these methods. Although the statistical results (Tables 4, 5; Figures 5–7) showed that these methods have achieved good prediction performance for DTA on commonly used benchmark datasets, the further improvement in DTA prediction still faces challenges. Researchers are actively working on extracting high-level implicit features from the sequences, structures, or complexes of drugs and targets, with the aim of developing novel methods with even better performance for predicting DTA.

**TABLE 4:** Performance comparison of multiple state-of-the-art methods based on PDBbind dataset.

Methods	MSE	RMSE	CI
Pafnucy	1.418	1.129	0.789
DeepDTA	1.443	1.148	0.771
DeepDTAF	1.355	1.073	0.799
FusionDTA	1.504	1.2	0.766
DataDTA	1.274	1.012	0.806
GraphDTA	1.579	1.193	0.66
WideDTA	1.633	1.295	0.638
DeepGS	1.385	1.096	0.784
DeepFusionDTA	1.235	1.203	0.774
AffinityVAE	1.398	1.102	0.792

**TABLE 5:** Performance comparison of multiple state-of-the-art methods based on KIBA and Davis datasets.

Dataset	Methods	MSE	CI	Methods	MSE	CI
KIBA	GanDTI	0.469	0.878	DGraphDTA	0.127	0.902
	GraphDTA	0.441	0.881	HiSIF-DTA	0.12	0.904
	WGNNDTA	0.43	0.886	GTAMP-DTA	0.123	0.917
	MGraphDTA	0.427	0.889	TransVAE-DTA	0.2536	0.8221
	UCMCDTA	0.421	0.891	AttentionMGT-DTA	0.14	0.893
Davis	GanDTI	0.236	0.885	DGraphDTA	0.202	0.905
	GraphDTA	0.225	0.895	HiSIF-DTA	0.191	0.907
	WGNNDTA	0.211	0.898	GTAMP-DTA	0.177	0.923
	MGraphDTA	0.205	0.899	TransVAE-DTA	0.3229	0.8596
	UCMCDTA	0.203	0.9	AttentionMGT-DTA	0.193	0.891

## 6 Conclusion

Deep learning-based computational methods for DTA prediction have become a crucial component of drug discovery in the pharmaceutical industry. Despite the significant progress achieved by these methods, there is still a gap between their current prediction accuracy and the expectations of researchers. Therefore, to further facilitate the development of novel and high-precision computational methods for DTA prediction, this review provides detailed statistics, summaries, and elaboration on commonly used datasets, the sequence and structural representations of drugs and targets, as well as representative deep learning methods.

From the comprehensive overview of advanced methods for predicting DTA based on deep learning, three key points stand out:

1. It is essential to thoughtfully combine deep learning models like CNN, RNN, and GNN to extract crucial implicit features influencing DTA prediction from the sequences, structures, and other data related to drugs and targets.
2. Deep learning models are employed to extract numerous features from diverse modalities of drugs and targets. Further refinement and effective fusion of these features are vital to obtain comprehensive deep features.
3. Most DTA prediction methods using deep learning lack explanations of their effectiveness. This absence hinders researchers from enhancing current methods.

In the future, it is imperative to delve into DTA prediction methods based on deep learning from three key perspectives:

1. Alongside commonly used deep learning models like CNN, RNN, and GNN, it is essential to incorporate unsupervised learning models such as contrastive learning to comprehensively capture the pivotal features influencing DTA prediction.
2. The emergence of tools such as AlphaFold has made it no longer difficult to obtain the structures of targets, with these structures playing a crucial role in determining molecular function. Hence, delving deeper into the three-dimensional spatial structural features of drugs and targets will help enhance the performance of DTA prediction.
3. While some deep learning-based methods for DTA prediction have shown promising results on standard datasets, their generalization performance is not satisfactory. Therefore, focusing on selecting specific datasets within particular fields and constructing deep learning models for DTA prediction that directly cater to practical application requirements will emerge as a prominent area of research interest.
